# Mental Health in Hispanic/Latino Older Adults With Multiple Chronic Conditions: Evidence and Recommendations

**DOI:** 10.1093/geroni/igaf122.056

**Published:** 2025-12-31

**Authors:** Irina Mindlis, Atami De Main, Daniel Jimenez

## Abstract

Among older adults with multiple chronic conditions (MCC), poor mental health increases the risk for morbidity and mortality. Hispanic/Latino older adults are more likely to sustain early onset of MCC and age in poverty, with poorer access to health care, and greater functional limitations. However, most research on mental health related to MCC has focused primarily on non-Hispanic/Latino populations. This symposium aims to highlight findings on the mental health of Hispanic/Latino older adults with MCC through a range of approaches, including in-depth interviews, community-based approaches, and national and cross-national studies. Camacho et al. address the assessment of loneliness and wellbeing narratives among Hispanic/Latino older adults with MCC and chronic pain, highlighting the importance of culturally-informed assessments. De Main et al. discuss barriers and facilitators to recruitment of this population for mental health research, in partnership with community-based organizations. Malatyali et al. present findings from the Health and Retirement Survey (HRS) on the disproportionate burden of poor psychological outcomes in Hispanic/Latino older adults with MCC. Finally, Bishop examines the comparability of depressive symptom indicators across the HRS and the Mexican Health and Aging Study, informing cross-national examinations of depressive symptoms and comorbidities among Mexican-origin older adults. Overall, these papers will provide insights and recommendations into issues of assessment, engagement, and best practices in the evaluation of mental health in Hispanic/Latino older adults with MCC. The findings will be discussed with attention to cultural and contextual factors, and highlight implications for future interventions to improve mental health outcomes in this community.

